# Prescription Cards

**Published:** 1895-02

**Authors:** 


### Prescription Cards. By Stacy Jones, M. D. Bœricke & Tafel, Publishers, 1011 Arch Street, Philadelphia.

These "cards" consist of little pamphlets of 16 pages, small octavo, containing a printed list of all the probable symptoms a patient is likely to have, together with a blank space for name of patient and the other personal items usually recorded.

Each symptom is followed by the name of the remedy which it indicates. These names are arranged after the manner of the *Cipher Repertory*, but the letters reversed, that the patient may not be able to read them.

The manner of using is that, the "card" being given to the patient, he or she draws a pencil mark under the symptoms complained of, and then, the prescription made being noted on the card, the latter is filed away for reference when the patient comes again. In this way there is kept a complete record of the patient's symptoms with a minimum of writing and book-keeping.

